# Slow-release praziquantel for dogs: presentation of a new formulation for echinococcosis control

**DOI:** 10.1186/s40249-017-0357-4

**Published:** 2017-09-15

**Authors:** Bin Jiang, Xiao-Nong Zhou, Hao-Bing Zhang, Yi Tao, Le-Le Huo, Ni Liu

**Affiliations:** 1 National institute of parasitic Diseases, Chinese Center for Disease Control and Prevention, 207 Ruijin Er Road, Shanghai, 200025 China; 2 Key Laboratory of Parasite and Vector Biology, Ministry of Health, 207 Ruijin Er Road, Shanghai, 200025 China; 3 National Center for International Research on Tropical Diseases, Ministry of Science and Technology, 207 Ruijin Er Road, Shanghai, 200025 China; 4 WHO Collaborating Centre for Tropical Diseases, 207 Ruijin Er Road, Shanghai, 200025 China

**Keywords:** *Echinococcus*, Transmission-blocking, Praziquantel, Enantiomer, In situ slow-release preparation, Subcutaneous injection, Pharmacokinetic, Stereoselective, Dog, China

## Abstract

**Background:**

Echinococcosis is a serious, zoonotic, parasitic disease with worldwide distribution. According to a epidemiological survey in 2012 in China, there are 20,000 infected patients and more than 50 million people at the risk. As the dog is the main, definitive host, the Government of China encourages monthly praziquantel treatment of every dog. However, this is difficult to achieve in geographically challenging areas, such as the Tibetan plateau, where there are also many dogs without owners. To overcome these problems, we investigated the transmission blocking capacity of a slow-release formulation of praziquantel administered by subcutaneous injection.

**Methods:**

The impact of a slow-release preparation of two pharmacokinetically stereoselective praziquantel enantiomers, i.e., R-(−)-praziquantel (R-PZQ) and S-(+)-praziquantel (S-PZQ) absorbed into a biodegradable polymer was studied in beagle dogs (*N* = 6). The preparation was given by subcutaneous injection using a single dose of 100 mg/kg. Chiral-selective, high-performance liquid chromatography (HPLC) and high-resolution mass spectrometry (HRMS) were applied to measure the praziquantel enantiomers in the plasma of the dogs. The lower limit for estimating plasma concentrations accurately for R-PZQ was 4 ng/ml and for S-PZQ 20 ng/ml. The pharmacokinetic parameters were calculated by a noncompartmental analysis model using Drug Analyze System (DAS) software 2.0. The SPSS 19.0 software was used for statistical analysis, and the statistical comparison between enantiomers was assessed using the two-tailed *t*-test.

**Results:**

Two hours after administration, peak concentrations of R-PZQ and S-PZQ: 321 ± 26 and 719 ± 263 ng/ml, respectively, were achieved. After 180 days, the average plasma concentration of R-PZQ in the six dogs had decreased to 13 ng/ml. The average concentration value of S-PZQ was higher than that of R-PZQ in the first 90-day period but fell afterwards and could not be accurately estimated when dropping below 20 ng/ml (the lower methodological limit for this enantiomer). Taking all the dogs into account, the average maximum concentration (C_max_) of S-PZQ in plasma over the first 3 months was higher than that of R-PZQ by 114.0% (*P* < 0.05), while the average mean retention time (MRT) of R-PZQ in plasma was higher than that of S-PZQ by 96.3% (*P* < 0.05).

**Conclusions:**

Praziquantel given as an in situ slow-release formulation by subcutaneous injection resulted in concentrations of the active principle in beagle dogs, which should be capable of resisting new *Echinococcus* infections for at least 6 months. The new formulation of praziquantel represents a potential, alternative way of presenting medication against tapeworm infections in dogs.

**Electronic supplementary material:**

The online version of this article (10.1186/s40249-017-0357-4) contains supplementary material, which is available to authorized users.

## Multilingual abstracts

Please see Additional file 1 for translations of the abstract into the five official working languages of the United Nations.

## Background

Echinococcosis (also called hydatid disease) is a serious, zoonotic, parasitic disease that exists as two main forms in humans: cystic echinococcosis and alveolar echinococcosis [1]. The adult *Echinococcus* worms reside in the small bowel of the definitive hosts (most often dogs and other canids- foxes and wolves) which pass the parasite eggs in the faeces. After ingestion by a suitable intermediate host (sheep, goat, swine, cattle, horse, camel and sometimes various rodents), the eggs hatch and release oncospheres that penetrate the intestinal wall and migrate to various organs, commonly liver and lungs, developing into cysts filled with protoscolices and daughter cysts that infect new definitive hosts when ingested. The protoscolices attach to the intestinal mucosa in the definitive hosts and develop into adults in 30 to 80 days. Humans become infected when living under low hygienic standards in close contact with infected dogs and this produces the same type of cysts in internal organs as seen in the infected intermediate hosts [2].

The disease is one of the now 18 neglected tropical diseases (NTDs) recognized by the World Health Organization (WHO) [3]. Its worldwide prevalence remains high and the estimated number of disability-adjusted life years (DALYs), a measure used to assess the burden of diseases [4], indicates that the impact of cystic echinococcosis must be taken very seriously. Highly endemic areas are common in the eastern part of the Mediterranean region, northern Africa, southern and eastern Europe, the southern tip of South America and Central Asia, including Siberia and western China [5, 6]. The estimated global burden of echinococcosis is 1.4 × 10^5^ DALYs [7].

According to a national survey in China in the endemic areas in 2005, the positive rate of echinococcosis was 12.04% (4796/39826) by serological examination with 1.08% cases confirmed by ultrasound [8]. Between 2004 and 2008, 10,790 new patients with echinococcosis were reported, mainly from the provinces of Sichuan, Xinjiang, Qinghai, Gansu, Ningxia and Inner Mongolia [9]. Sichuan Province reported 10,037 additional cases between 2007 and 2012 resulting in an average of 807 new cases per year [10].

According to a recent estimate of the total Chinese market, the demand for veterinary antiparasitic drugs (2013) was about CNY 4 billion, CNY 1.5 billion of which represent drugs against cestodes and trematodes [11]. Based on these figures, the annual sale could reach CNY 800 million for the new formulation of praziquantel (PZQ) and diagnostic reagent needed to protect all people and livestock at risk in the endemic areas. Introduction of better echinococcosis control would not only make life better for people threatened by the infection, but also save about CNY 3 billion of economic losses incurred in the food industry. Indeed, the financial burden of cystic echinococcosis of global, animal health costs was growing at a rate of US$ 2 × 10^9^ annually [12].

Anthelmintic treatment of owner dogs using PZQ [13–16] is a recommended control procedure in various countries [17–20], but the difficulty in effectively blocking transmission has slowed progress, and it can take decades to eliminate echinococcosis in the endemic areas. The National Echinococcosis Control Programme of China is conducting an approach where every dog with a known owner is treated with PZQ once every month, which should break the *Echinococcus* life cycle in principle [21, 22]. In the Tibetan area, however, this strategy does not work well, the many dogs without owners being part of the problem [21, 23]. In addition, frequent dog treatment is not only time-consuming but also costly. Extending the treatment interval may be necessary in economically resource-poor countries, or in areas where there are severe logistical constraints as on the Tibetan Plateau. In such cases, it would be essential to combine periodic anthelmintic treatment with other approaches,such as vaccination of sheep [24, 25]. However, the strategy to treat only 1–2 times per year, which was used in the period 1986–1991, led to strong environmental contamination by *Echinococcus* eggs, which was shown in Xiaoguai Village, Karamay City and the Xinjiang Uygur Autonomous Region [26]. Also in Turkana, Kenya, this approach failed to control reinfection of dogs when only 59% of all registered dogs were treated [27]. To overcome these shortcomings, new delivery strategies, such as PZQ-loaded hydrogenated castor oil solid lipid nanoparticles [28] and various forms of slow-release PZQ implants, have been tried [29, 30]. However, these strategies have side effects, e.g., the former approach only releases PZQ for 13 days [28] and therefore needs to be frequently repeated, while implants require surgical intervention that carries risk of infection.

PZQ is a racemic mixture of two enantiomers, R-(−)-praziquantel (R-PZQ) and S-(+)-praziquantel (S-PZQ) [31, 32], the former being the main effector with the latter and its metabolites not having significant antiparasitic properties [33–38]. While the comparatively high price of producing pure R-PZQ limits its application in practice, there are enantio-selective, metabolic pathways with regard to R-PZQ and S-PZQ that could be useful in a future scenario [39]. These pathways consist of oxidation, dehydrogenation and glucuronidation involving three catalytic enzymes of the cytochrome P450 family, CYP3A, CYP2C9 and CYP2C19, which act differently with respect to the two PZQ enantiomers [40]. Computer docking models have shown that both enantiomers enter the same site on the enzyme but that R-PZQ may exhibit a better fit [40]. The importance of R-PZQ is emphasized by the fact that research focused on improved production of this enantiomer has been designated a key priority by the UNICEF/UNDP/World Bank/WHO Special Programme for Research and Training in Tropical Diseases (TDR) [41].

In light of the discussion above, it was felt to be important to follow the presence of both enantiomers from a temporal point of view. We used the dog as the experimental animal and used a biodegradable drug/matrix preparation allowing the PZQ to be released in a sustained way as Eligard^®^ [42, 43]. For the detection of the enantiomers, we tried a chiral method (i.e., based on molecular, assymetric properties) for PZQ enantiomer selection using high-performance liquid chromatographic (HPLC) and high-resolution mass spectrometry (HRMS) [44, 45]. The objectives of this study were to investigate the pharmacokinetics of R-PZQ and S-PZQ and the effect of the new drug formulation analysing the release characteristics in vivo for future application in a field trial aimed at echinococcosis control based on treatment of the definitive host.

## Methods

Aliquots of standardized R-PZQ, S-PZQ and mebendazole were first tested for validation. Then, plasma samples from the dogs, followed over 6 months after injection of the slow-release polymer/drugs preparation, were investigated. The mebendazole standard was used in all plasma samples from the dogs as internal standard (IS) to avoid potential errors due to sample preparation and/or injection volume.

### Chemicals and materials used

PZQ standard used for methodological analysis was purchased from FLUKA (Shanghai, China), while the R-PZQ and S-PZQ standards were purchased from TLC Pharmaceutical Standards (Shenzhen, China). PZQ for subcutaneous administration was purchased from Nanjing Pharmaceutical Factory (Nanjing, China). Mebendazole used as internal standard (IS) was purchased from National Institute for Food and Drug Control (Beijing, China).

HPLC-grade formic acid was purchased from Dikma Technologies Inc. (Beijing, China). HPLC-grade methanol and acetonitrile were purchased from Emerck, Sinopharm Chemical Reagents Co., Ltd. (Shanghai, China). Water was purified using a RODI-068598 Hitech-kflow purification system (Merck Millipore, Milford, MA, USA). OASIS^®^ HLB SPE 96-wells plates (30 mg) and OSTROTM 96-wells plates were purchased from Waters (Shanghai, China). All chemicals and solvents used were of analytical grade.

Materials used for the production of the slow-relaease preparation, see below under this heading.

### HPLC–HRMS equipment and conditions

The HPLC–HRMS chromatographic system included an Accela 1250 pump, a PAL HTC autosampler, an Accela PDA detector and a Xcalibur data system (ThermoFisher Scientific, Waltham, MA, USA). Water was purified by an Ultra Clear system (Siemens, Berlin, Germany). Chromatographic separation of R-PZQ, S-PZQ and IS mebendazole were obtained on a column of Daicel  CHIRALPAK^®^ AS-RH (4.6 mm × 150 mm, 5 μm) fitted with a guard column (10 × 4.0 mm, 3 μm) at room temperature (about 25 °C). The autosampler was set at 4 °C during analysis. The mobile phase consisted of methanol and formic acid (90:10, v/v) at the flow rate of 0.55 ml/min. The relative parameters were as follows: ESI ion source, 3.00 kV spray voltage, sheath gas (N_2_) and auxiliary gas (N_2_) 35 Arb and 10 Arb, respectively, capillary heater temperature 320 °C. The [M -H]^+^ quasi-molecular ion peaks of R-PZQ and S-PZQ were detected at 313.1911 mass-to-charge (m/z) ratio (the two enantiomers have the same molecular weight and molecular structure and differ only with regard to symmetry), while the IS (mebendazole) was detected at 296.1036 m/z. The total run time was 25.0 min.

### Standards and quality control

Stock solutions of R-PZQ and S-PZQ standard were prepared for the establishment of calibration curves of R-PZQ and S-PZQ. Stock solutions of R-PZQ and S-PZQ were prepared by dissolving 5 mg of compound in 10 ml methanol, respectively. Standard solutions were prepared by serial dilution of the stock solution with methanol. An IS solution of mebendazole was prepared by dissolving 5 mg mebendazole in 1 ml formic acid, then diluted to 10 ml by methanol at a concentration of 500 μg/ml. All solutions were stored at 4 °C until use.

For the establishment of the PZQ calibration curve, 0.5 ml blank dog plasma samples with diluted PZQ stock solution at the concentrations of 4, 10, 20, 40, 80, 160, 400, 800, 1600, 2000, 3200 and 10,000 ng/ml were extracted as described in the Sample Preparation section above.

### Extraction recovery and chromatography

R-PZQ and S-PZQ at low (40 ng/ml), medium (400 ng/ml) and high (3200 ng/ml) concentrations were added to 0.5 ml of blank dog plasma samples for extraction as described in the Sample Preparation section without adding the internal standard (IS). Then the residue was dissolved by methanol with 10 μl IS, followed by filtration through a 0.22 μm filter membrane. The Rr (Rr = Ar/Ai) and Rs (Rs = As/Ai) of each drug concentration were calculated according to the drug peak areas (Ar and As) and the IS peak area (Ai). The Rr’ (Rr’ = Ar′/Ai’) and Rs’ (Rs’ = As′/Ai’) of each drug concentration were calculated according to the drug peak areas (Ar′ and As′) and the IS peak area (Ai) by the same concentrations of R-PZQ, S-PZQ standard and IS without extraction. The absolute extraction recoveries of R-PZQ and S-PZQ were obtained by Rr/Rr’ × 100% and Rs/Rs’ × 100%, respectively. Liquid aliquots of 3 μl of material generated by the extraction procedure were assayed by HPLC–HRMS (on the same day) five times and also over 5 days to obtain the within-day as well as between-day precision.

### Animals

Six beagle dogs, weighing between 8 and 10 kg, were provided by the Agricultural College of Shanghai Jiao Tong University (Shanghai, China). Immediately before being used in the study, the dogs were kept fasting for 12 h with free access to water before any experiments were carried out. All animals were handled in accordance with the guidelines for animal care of the National Institute of Parasitic Diseases (NIPD)at Chinese Center for Disease Control (CDC), Shanghai, and the Guide for the Care and Use of Laboratory Animals (2011). The study was approved by the NIPD Institutional Ethics Committee for Animals.

### Slow-release PZQ preparation

A stock preparation was made by adding 16 wt% matrix poly D,L-lactide-co-glycolide (PLGA) precursor to 51 wt% N-methyl-2-pyrrolidone solvent. This solution was mixed with 33 wt% racemic PZQ and drug amounts corresponding to 100 mg/kg per dog were immediately injected subcutaneously on the back of each dog, where it rapidly coagulated into a biodegradable polymer from which the drug diffused in a sustained way during several months as described by Singh et al. [43].

### Dosing and sample collection

The six dogs were all given a single dose of the racemic mixture (R-PZQ + S-PZQ) together with the slow-release polymer precursor by subcutaneous injection using a 16G syringe needle gauge. The amount administered to each dog was based on weight giving 100 mg/kg. Approximately 1 ml blood samples were collected in heparinised vacuum tubes from the jugular vein of the dogs using 7G needles 2 h after the single subcutaneous injection and then on day 3, 10, 20, 30, 40, 50, 60, 90, 120 and 180. The blood samples from each dog immediately centrifuged at 3000 r/min for 10 min, the plasma then removed and stored at −20 °C.

### Sample preparation

For extraction of PZQ from plasma, the frozen plasma samples were thawed and 0.5 ml water was added to the plasma and mixed using vortex during 1 min. Then, 10 μl of the mebendazole IS solution (500 μg/ml), 20 μl of 0.1 M NaOH and 10 μl of formic acid were added successively and mixed by vortex for 30 s. after each addition. Subsequently, the plasma samples were subjected to solid phase extraction (SPE) on a Waters C-18 Column. In each column, the cartridge was equilibrated with 2 ml of methanol and 2 ml of deionized water prior to adding the sample. After it had been loaded onto the cartridge, 2 ml of 5% methanol was used for washing followed by the addition of 1.2 ml of 10% formic acid in methanol to elute any PZQ into a new test tube, the contents of which were completely evaporated under a gentle stream of nitrogen at 50 °C. The residue was reconstituted with 1000 μl of methanol and filtered by a 0.22 μm filter membrane and an aliquot of 3 μl of the resulting solution was used for the chiral HPLC-HRMS analysis.

### Statistical analysis

The pharmacokinetic parameters such as the maximum concentration (C_max_), the area under the curve (AUC), the average time required to decrease to 50% of the original concentration level (T_1/2_), mean retention time (MRT) and the time of maximum concentration (T_max_), were calculated by a noncompartmental analysis model using Drug Analyze System (DAS) software 2.0 and expressed as mean ± standard deviation (SD) (*P* < 0.05). The SPSS19.0 software was used for statistical analysis, and the statistical comparisons between enantiomers after administration were assessed using the two-tailed *t*-test.

## Results

### Method validation

**Fig. 1:**
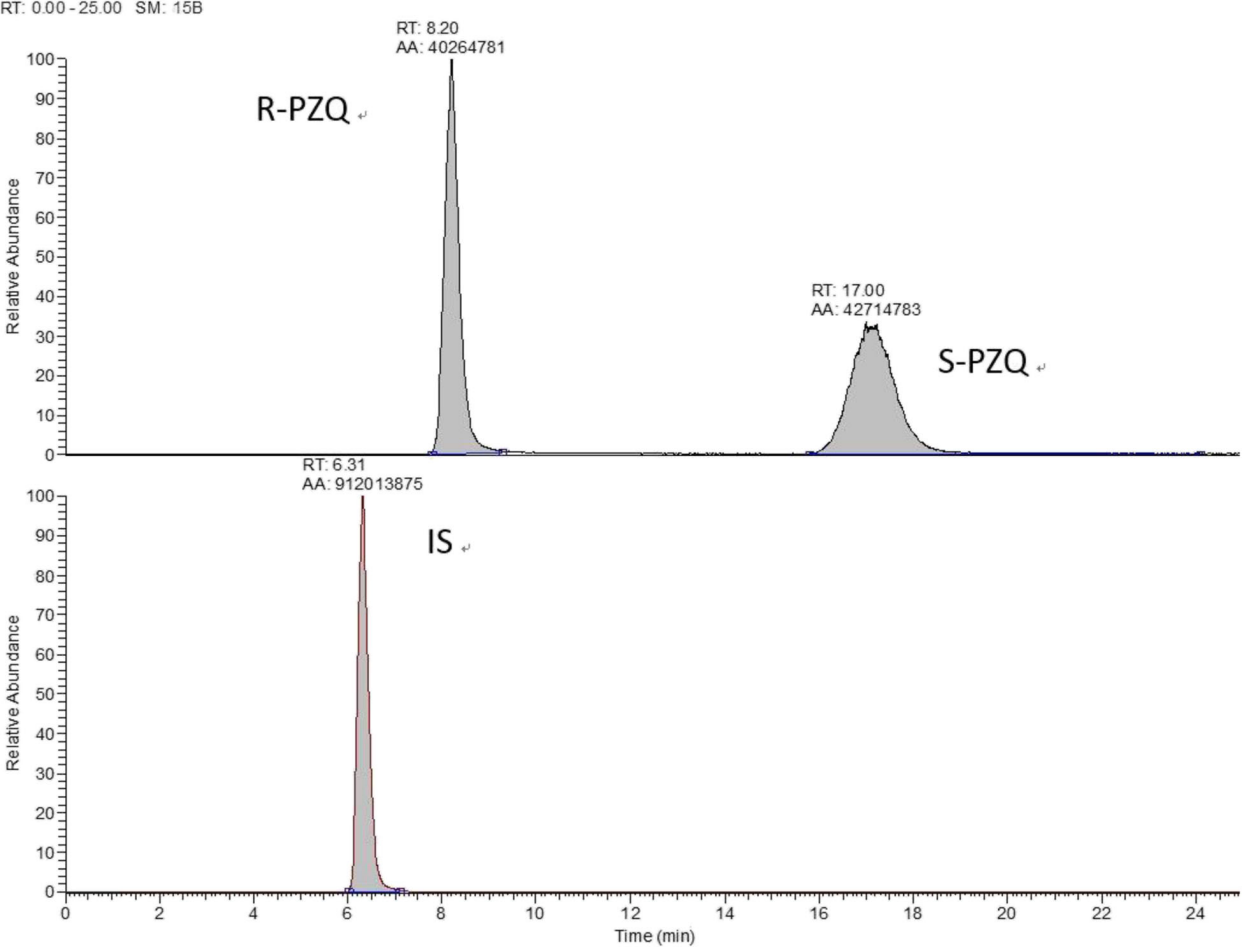
Methodological validation of the HPLC-HRMS approach using standard preparations of R-PZQ, S-PZQ and mebendazole in blank plasma. Smoothed curves (SM) resulting after a total running time (RT) of 25.00 min; RT = retention time at peak of the signal; AA = area under the curve; mebendazole was used as internal stanard (IS). The mass-to-charge ratio (m/z) made it possible to calculate the mass of the compounds and thus identify the findings in each run: R-PZQ (313.1880–313.1942), S-PZQ (313.1880–313.1942) and mebendazole (296.1000–296.1060). R-PZQ was faster than S-PZQ so ion peaks of R-PZQ were in the first line

**Fig. 2:**
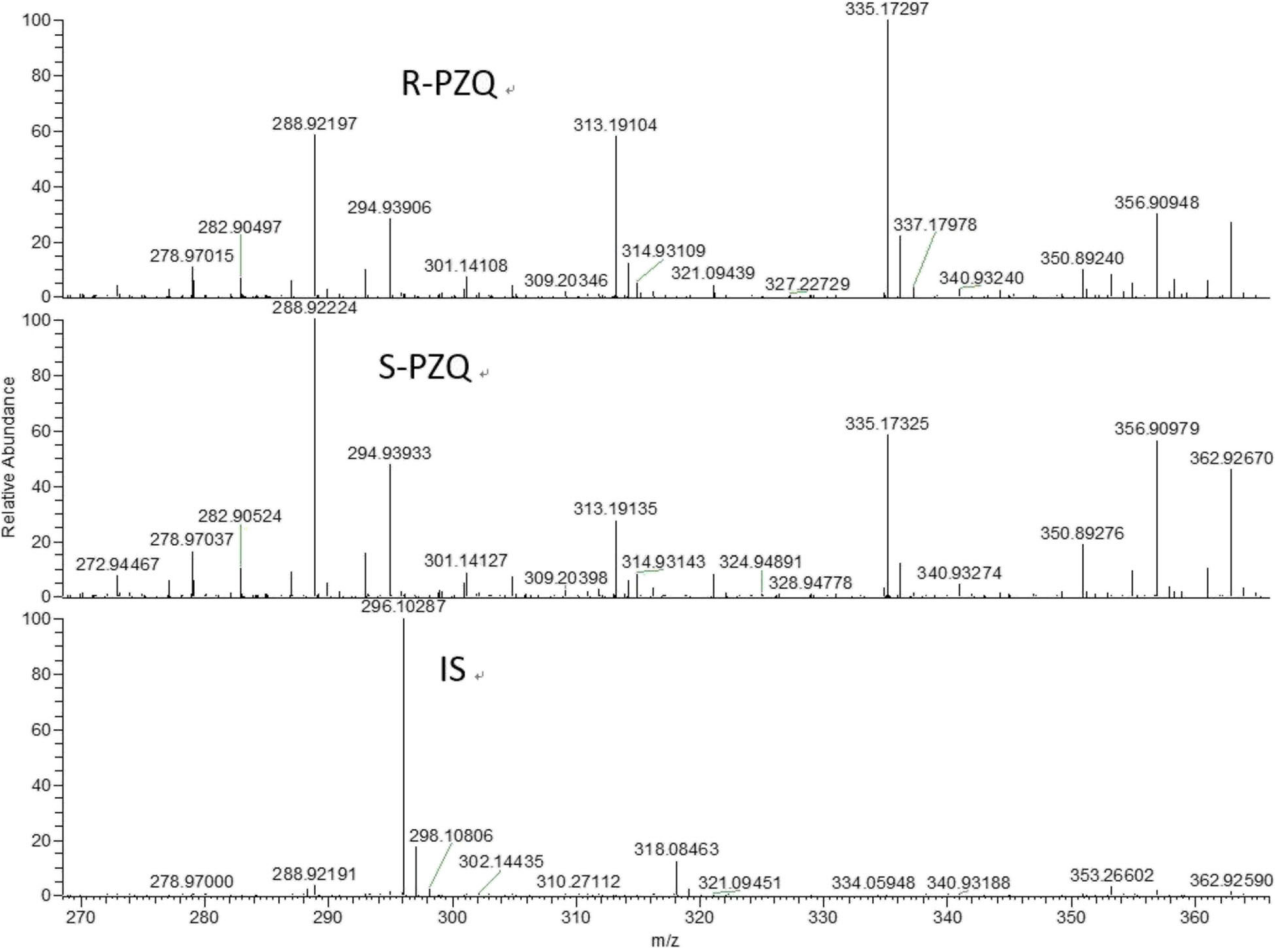
Full scan mass spectra of R-PZQ, S-PZQ and mebendazole (IS) of standard solutions in blank plasma showing molecular ion peaks and fragment ion peaks of these compounds. The figures given for the ion peaks were the mass-to-charge ratios (m/z), i.e., signature outcomes for each compound, which made it possible to identify all constituents

**Fig. 3:**
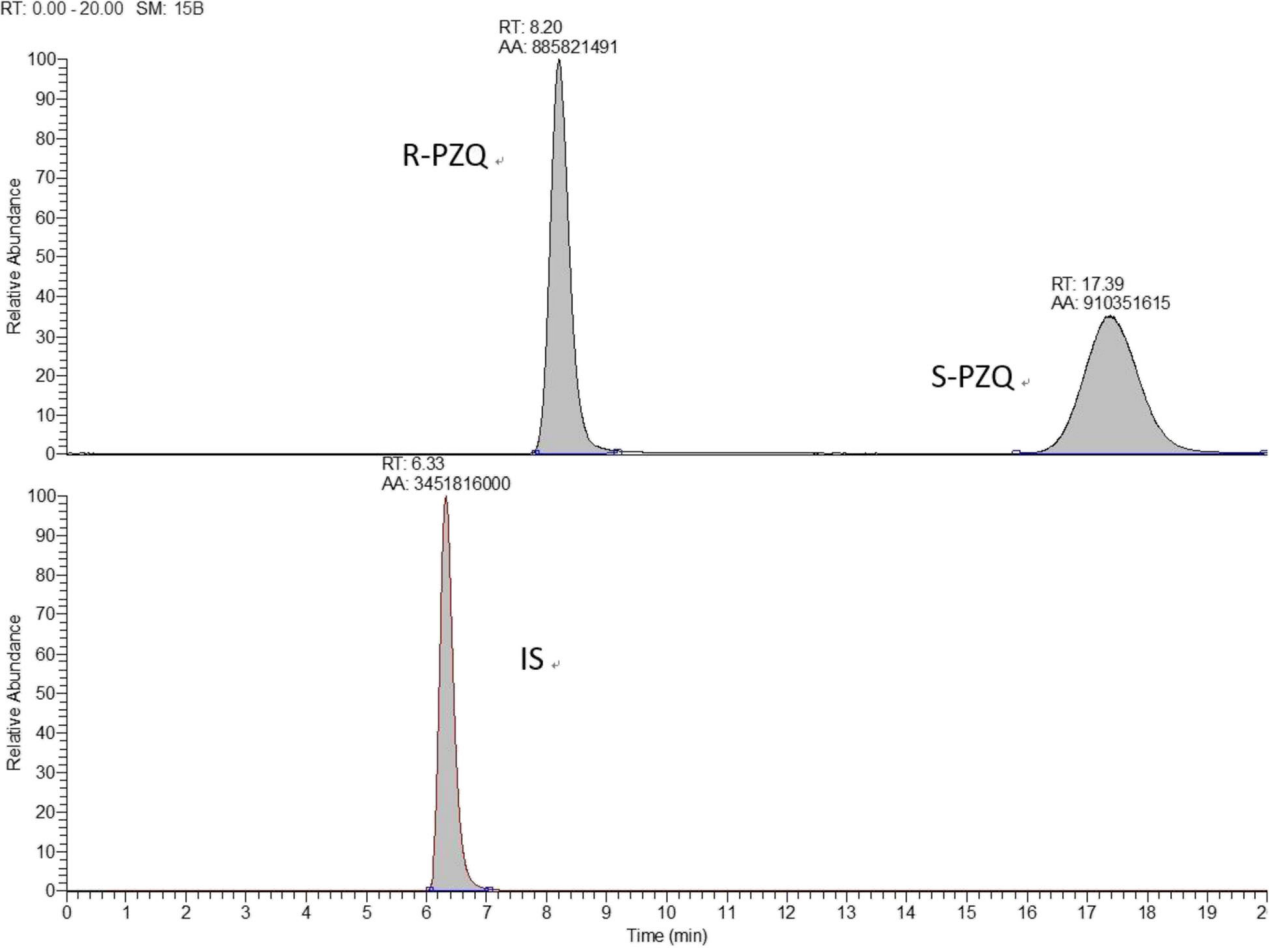
Results of one HPLC-HRMS run of one dog plasma sample taken after the administration of 100 mg/kg of the new slow-realease formulation. Smoothed curves (SM) resulting after a total running time (RT) of 20.00 min; RT = retention time at peak of the signal; AA = area under the curve; mebendazole was used as internal stanard (IS). Identification of the compounds as R-PZQ, S-PZQ and mebendazole (the internal standard) was made possible by calculations based on the mass-to-charge ratio (m/z) values

The chiral HPLC method applied for separation of the enantiomers worked very well and the HRMS determination process was suitably sensitive for the detection of PZQ and the IS (mebendazole) (Figs. 1 and 2). Both enantiomers had resolved from the matrix and diffused into the plasma well with the two enantiomers clearly separated from each other (Fig. 3).

The calibration curve of R-PZQ was constructed by plotting the plasma concentrations of R-PZQ (Cr) from 4 to 10,000 ng/ml against the drug peaks and the IS peak area ratios (Rr). For determining the plasma concentration, the regression equation of the calibration curve using the weighted least-squares linear regression method and its correlation coefficient *r* were as follows: Cr = 59 643Rr (*r* = 0.9993). The calibration curve of S-PZQ was constructed by plotting the plasma concentrations of S-PZQ (Cs) from 20 to 10,000 ng/ml against the drug peaks and the internal standard peak area ratios (Rs). For determining the plasma concentration, the calibration curves of S-PZQ was linear, while the regression equation of the calibration curve and its correlation coefficient *r* were as follows: Cs = 54 259Rs (*r* = 0.9986). The limits of quantification for R-PZQ or S-PZQ were 4 ng/ml and 20 ng/ml, respectively.

The mean absolute extraction recoveries for the low, medium, and high concentrations of R-PZQ were (93.0 ± 7.3) %, (94.6 ± 8.8) % and (86.8 ± 5.3) %, respectively (Table 1). The corresponding concentrations of S-PZQ were (87.1 ± 8.0) %, (91.9 ± 7.2) % and (86.1 ± 4.5) %, respectively (Table 1). The mean relative recoveries for the low, medium, and high concentrations of R-PZQ were (95.0 ± 2.9) %, (86.3 ± 2.6) % and (89.0 ± 5.2) %, respectively (Table 2). The corresponding concentrations of S-PZQ were (88.5 ± 0.6) %, (84.8 ± 3.4) % and (88.6 ± 5.2) %, respectively (Table 2).

**Table 1:** Results of the absolute extraction recovery tests

Concentration(ng/ml)	R-PZQ			S-PZQ		
	40	400	3200	40	400	3200
First run	100.1	93.3	81.2	96.3	89.7	83.5
Second run	93.2	104.0	87.3	83.3	99.9	91.4
Third run	85.5	86.5	91.8	81.7	86.0	83.5
Recovery (%)	93.0 ± 7.3	94.6 ± 8.8	86.8 ± 5.3	87.1 ± 8.0	91.9 ± 7.2	86.1 ± 4.5

**Table 2:** Results of the relative extraction recovery tests

Concentration(ng/mL)	R-PZQ			S-PZQ		
	40	400	3200	40	400	3200
1	96.3	87.3	89.6	88.2	83.7	94.2
2	91.8	88.3	83.5	88.0	88.6	87.5
3	97.1	83.4	93.8	89.2	82.2	84.0
Recovery (%)	95.0 ± 2.9	86.3 ± 2.6	89.0 ± 5.2	88.5 ± 0.6	84.8 ± 3.4	88.6 ± 5.2

The within-day relative standard deviations (RSDs) for low, medium, and high concentrations of R-PZQ were 3.3%, 1.1% and 2.3%, respectively; and the corresponding between-day RSDs were 6.9%, 5.6% and 5.3%, respectively (Table 3). The within-day RSDs for low, medium, and high concentrations of S-PZQ were 7.3%, 0.9% and 1.7%, respectively; and the corresponding between-day RSDs of S-PZQ were 10.8%, 5.7% and 5.5%, respectively (Table 3). As can be seen in Table 3 the precision of within-day sample readings and between-days readings, no RSD showed a variation exceeding 15%.

**Table 3:** Precision of Sample within-day and between-day (*n* = 5)

Concentration(ng/mL)	R-PZQ RSD^a^ (%)		S-PZQ RSD^a^ (%)	
	Within-day^b^	Between-day^b^	Within-day^b^	Between-day^b^
40	3.3	6.9	7.3	10.8
400	1.1	5.6	0.9	5.7
3200	2.3	5.3	1.7	5.5

^a^Relative standard deviation; ^b^ *N* = 5

### Pharmacokinetics of PZQ in situ slow-release subcutaneous injection and its enantiomers

#### Plasma samples

**Fig. 4:**
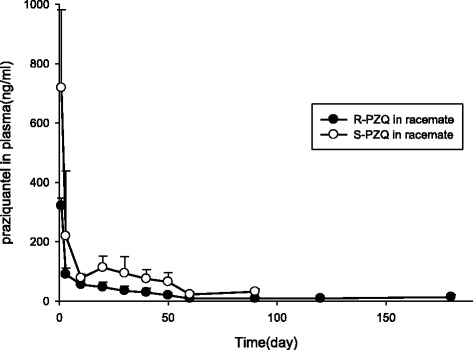
Change over time of R-PZQ, S-PZQ and racemic PZQ concentration in dog plasma after the administration of 100 mg/kg of the new slow-release formulation. Plasma samples taken after 2 h and at day 3, 10, 20, 30, 40, 50, 60, 90, 120 and 180

The plasma concentration–time curves of R-PZQ, S-PZQ and PZQ measured are shown in Fig. 4. After subcutaneous administration of racemic PZQ by in situ slow-release subcutaneous injection at a 100 mg/kg for 2 h, the highest PZQ concentrations were (321 ± 26) and (719 ± 263) ng/ml for R-PZQ and S-PZQ, respectively. R-PZQ decreased to the 13 ng/ml after 180 days. The average concentration value of S-PZQ was higher than that of R-PZQ in the first 90-day period but fell afterwards and could not be accurately estimated when dropping below the lower methodological limit (20 ng/ml).

After subcutaneous administration of praziquantel sustained-released subcutaneous injection, the main pharmacokinetic parameters of R-PZQ and S-PZQ at the single dose of 100 mg/kg is shown in Table 4 and could be described as follows: R-PZQ, the T_max_ was (2 ± 0) h, C_max_ was (321 ± 26) μg·l^−1^, T_1/2_ was (2025 ± 1458) h, the MRT was (1180 ± 374) h, AUC was (92,240 ± 14,546) μg·l^−1^·h; S-PZQ, the T_max_ was (2 ± 0) h, C_max_ was (719 ± 263) μg·l^−1^, T_1/2_ was (1003 ± 406) h, MRT was (601 ± 106) h, AUC was (165,348 ± 31,359) μg·l^−1^·h. The average AUC value of S-PZQ in plasma was higher than that of R-PZQ by 79.2% (*P* < 0.01). The average C_max_ value of S-PZQ in plasma was higher than that of R-PZQ by 114.0% (*P* < 0.05). The average MRT value of R-PZQ in plasma was higher than that of S-PZQ by 96.3% (*P* < 0.05). The average T_1/2_ of R-PZQ was higher than that of S-PZQ by 101.9%.

**Table 4:** Pharmacokinetic parameters in plasma after a single 100 mg/kg dose of the new PZQ slow-release formulation

Formulation	Dose(mg/kg)	T_max_/h	C_max_/μg·l^−1^	T_1/2_/h	MRT/h	AUC/μg·l^−1^·h
R-PZQ	50	2 ± 0	321 ± 26	2025 ± 1458	1180 ± 374	92,240 ± 14,546
S-PZQ	50	2 ± 0	719 ± 263*	1003 ± 406	601 ± 106*	165,348 ± 31359**

Average of results based on plasm from beagle dogs (*N* = 6); data are expressed as mean ± SD; the AUC values zero to time “t”; * *P* < 0.05; ** *P* < 0.01 vs. R-PZQ (“*” refers to significant difference when S-PZQ was compared with R-PZQ using the two-tailed *t*-test)

## Discussion

R-PZQ is known to be the biologically active enantiomer molecule against various parasites [33–38], but metabolic data of racemic PZQ in dogs have not been previously reported, something which is important when attempting to break the *Echinococcus* life cycle, as this animal is the most common definitive host for this parasite. The enantio-selectivity in the metabolism of R-PZQ and S-PZQ in Beagle dogs has been elucidated, and we report the usefulness of a new in situ slow-release PZQ preparation for transmission blocking of this parasitic disease.

Our experience during the experiments was that the PLGA polymer that had been injected subcutaneously degrades slowly and fed praziquantel into the dogs’ plasma over several months. Although PLGA is known to eventually hydrolyse into the non-harmful original monomers lactic acid and glycolic acid [44], we are well aware that much more detail, such as standardized PZQ incorporation into PLGA and its long-term stability at the dogs’ body temperature as well as minimum plasma levels and sensitivity will be needed.

The S-PZQ plasma concentration values were always larger than the R-PZQ ones in the first 3 months with the R-PZQ/S-PZQ ratios decreasing from 0.72 to 0.28 over this time, which was similar to the serum ratios (range: 0.54 to 0.33) previously measured in five healthy Caucasian volunteers by Westhoff et al. [44]. These results confirm that PZQ enantiomers are subject to selective metabolism similar to the enantio-selective activity noted against the adult schistosome worm [32–36]. The initial average AUC and C_max_ value of S-PZQ in plasma were higher than that of R-PZQ by 79.2% (*P* < 0.01) and 114.0% (*P* < 0.05), respectively, which meant that the AUC_R/S_ ratio of dogs was 0.56, while this ratio was 0.39 in a healthy volunteer [45]. As seen in Table 4, the results were similar in silico, in vitro, and in vivo, revealing the enantio-selective metabolic profile of PZQ due to the different metabolic pathways between R-PZQ and S-PZQ shown by Wang et al. [40]. As reported by Xiao et al. [37], the statistical medians (of the ranges) of T_1/2_ in plasma across nine patients, each given 25 mg/kg PZQ orally three times with 4-h intervals, were 1.1 (1.0–3.0) hours and 3.3 (1.9–3.9) hours for R-PZQ and S-PZQ, respectively. As seen in our experiments (Table 4), the average values of T_1/2_ and MRT for R-PZQ were higher than those recorded for S-PZQ by 101.9% and 96.3% (*P* < 0.05), respectively, supporting the idea that S-PZQ was more rapidly metabolized and disappears from the circulation faster in non-infected subjects. Alternatively, that the R-PZQ release time was longer than that of S-PZQ for the new formulation, which was supported by other reports suggesting possible stereo-selective release of racemic drugs from polymers in vivo [46, 47]. With R-PZQ being the effective enantiomer, this would result in a longer therapeutical activity.

Subcutaneous injection of PZQ has been shown to be more effective than the oral and intramuscular routes as it not only enhances bioavailability but, importantly, increases the systemic circulation time [28, 48]. The dose of 5 mg/kg eliminates 100% of immature adult *Echinococcus* parasites in dogs [49] and subcutaneous slow-release PZQ implants carrying doses of 36.4–66.7 mg/kg have been shown to prevent challenge infection with *Echinococcus granulosus* protoscolices up to 4 months [50]. In this study, the highest PZQ concentrations were reached 2 h after administration of 100 mg/kg PZQ using in situ slow-release preparation with R-PZQ still at 13 ng/ml after 6 months. Anti-schistosomal activity of PZQ was not only related to C_max_ but also to the duration of exposure [35], and exposure of tapeworms to high drug concentrations and/or long time was essential for adequate treatment [51, 52]. The mortality rate and lethal concentration of racemic PZQ on *Echinococcus* adult worms in vitro, was found to be 17.77 ng/ml, which meant that IC_50_ of R-PZQ was 8.89 ng/ml [53]. At the dose of 5 mg/kg of PZQ-loaded hydrogenated castor oil solid lipid nanoparticles, *E. granulosus* fecal egg excretion disappeared completely from dogs treated experimentally compared to the control group with C_max_ reported as 113.7 ng/ml of racemic PZQ, decreasing to near 10 ng/ml by 96 h [28]. Considering the concentration and release time of R-PZQ, we feel that the new in situ slow-release formula protects dogs against *Echionococcus* infection for at least 6 months. In addition, the slow-release approach as used here has several advantages: (1) the pain and infection risk of surgical implantation is avoided; (2) the drug lasts for months, which simplifies administration and reduces adverse effects; (3) simple formulation preparation and convenient dosage control; and (4) removal of polymer residues is not required as it is biodegradable.

Control programmes in three countries/islands (New Zealand, Tasmania and Cyprus) have achieved eradication within 10–12 years by policies focusing on the dog definitive host through control, especially using humane euthanasia [18]. In the Tibetan area of China, however, the religion is strongly Buddhist and it forbids culling animals, including dogs without an owner [54]. The Chinese National Echinococcosis Control Programme has resorted to treat all dogs with PZQ on a monthly basis, based on the experience in the Falkland Islands as well as Argentina and Chile to substantially reduce the prevalence of echinococcosis without reducing the dog population [55, 56]. However, this approach is not entirely successful due to the need to sustaining the periodic treatment for the long term [26] and including a high rate of the dog population [27]. According to mathematical modelling, however, it should be possible to block *Echinococus* transmission at, a PZQ treatment interval of at least 3 months and reach at least 60% of all dogs [57]. Prolonging the treatment period to over 6 months by the new in situ PZQ slow-release formulation would be sufficient to effectively block echionococcosis transmission, especially in resource-poor areas, such as the Tibetan Plateau. However, its usefulness now needs to be tested in a field trial in dogs infected with *Echinococcus parasites* to comfirm its efficacy.

Elimination of echinococosis is on of the Sustainable Development Goals (SDGs) [58] and PZQ is on WHO’s List of Essential Medicines [59], which means that it has a wide safety margin and the 50% lethal dose (LD_50_) of PZQ for dogs is over 200 mg/kg per os and 3000 mg/kg by subcutaneous injection [60]. Indeed, dogs have been given up to 180 mg/kg of PZQ orally for 13 weeks without organ damage [61] and single doses varying between 36 and 67 mg/kg in other dogs, sustained within 897–3685 ng/ml PZQ levels in serum after slow-release PZQ implants, without any liver and kidney abnormalities [50]. Considering that all PZQ plasma concentrations in our experiments were lower than those referred to above, we did not feel that a pathological evaluation of the dogs was warranted.

## Conclusions

Treatment of dogs by PZQ given as in situ sustained-release formulation by subcutaneous injection should be capable of resisting new *Echinococcus* infections for over 6 months in beagle dogs. The new formulation is a potentially alternative technique to provide effective medication against tapeworm infection. The new PZQ formulation represents not only as treatment for tapeworm infections, but is also a way to block transmission by preventing environmental pollution of the helminth eggs.

## Additional files

Additional file 1: Translation of the abstract into the five official working languages of the United Nations. (PDF 653 kb)

## Abbreviations

AUC: Area under the curve
C_max_: Maximum concentration
DALYs: Disability adjusted life years
DAS: Drug analysis system
HPLC: High-performance liquid chromatography
HRMS: High-resolution mass spectrometry
IC_50_: Half maximal inhibitory concentration
IS: Internal standard
MRT: Mean retention time
NTDs: Neglected tropical diseases
PZQ: Praziquantel
R-PZQ: R-(−)-praziquantel
RSD: Relative standard deviation
SDGs: Sustainable Development Goals
S-PZQ: S- (+)-praziquantel
WHO: World Health Organization
