# Supplementary material for: Slow-release praziquantel for dogs: presentation of a new formulation for echinococcosis control
Source: Infect Dis Poverty. 2017 Sep 15;6:140. doi: 10.1186/s40249-017-0357-4 (PMC5599885; doi:10.1186/s40249-017-0357-4)
Supplement: Supplementary file 1 — Translation of the abstract into the five official working languages of the United Nations. (PDF 653 kb) [file 40249_2017_357_MOESM1_ESM.pdf]

Translation of the abstract into the six official working languages of the United Nations

الإطلاق البطئ للمعالجة المشتركة بالبرازكوانتيل الكلاب: عرض تقديمي لتركيبة جديدة المشوكات التحكم

بن جيانغ، تشو شياو نونغ، وهاو بينغ تشانغ، يي تاو، لي لي هوة، ني ليو

ملخص

خلفية: المشوكات هو مرض خطير،طفيلي، وحيواني المنشأ انتشر في جميع أنحاء العالم. وفقا لدراسة وبائية 2012 في الصين، وجد عدد المرضى المصابين 20000 وأكثر من 50 مليون شخص في خطر. والكلب هو المضيف الرئيسي للمرض، وتشجع "حكومة الصين" العلاج الشهري بالبرازكوانتيل لكل كلب. ومع ذلك، وهذا أمر يصعب تحقيقه في وجود التحديات الجغرافيا مثل هضبة التبت، حيث يوجد أيضا العديد من الكلاب الضالة. للتغلب على هذه المشاكل، ونحن نحقق في امكانية حظر انتقال صيغة الإطلاق البطيئة للمعالجة المشتركة بالبرازكوانتيل عن طريق الحقن تحت الجلد.

أساليب: أثر إعداد بطيء-الإفراج عن اثنين فارماكوكينيتيكالي تراكما أحيائياً فراغي النوعية للمعالجة المشتركة بالبرازكوانتيل متبلور مضاد، أي آر-(-)-المعالجة المشتركة بالبرازكوانتيل (R-بزق) و S-(+)-درس المعالجة المشتركة بالبرازكوانتيل (S-بزق) استيعابهم في بوليمر القابلة للتحلل في الكلاب بيغل (ن <bx/> ). اخذت الاحتياطات عن طريق الحقن تحت الجلد باستخدام جرعة واحدة من 100 مجم/كجم. وطبقت مراوان الانتقائي، وعالية الأداء اللوني السائل ([هبلك]) واستبانة الطيف الكتلي لقياس المعالجة المشتركة بالبرازكوانتيل متبلور مضاد في بلازما الكلاب. الحد الأدنى لتقدير تركيزات البلازما بدقة للبحث والتطوير-بزق كان 4 نانوغرام/مليلتر وبزق S 20 نانوغرام/مليلتر. بنموذج تحليل نونكومبارتمينتال باستخدام برنامج نظام تحليل المخدرات (DAS 2.0) حساب بارامترات الحرائك الدوائية. تم استخدام البرمجيات SPSS 19.0 للتحليل الإحصائي، وتم تقييم المقارنة الإحصائية بين متبلور مضاد استخدام اثنين-الذيل تي-test.

نتائج: ساعتين بعد الإدارة، ذروة تركيزات بزق R و S-بزق: نانوغرام/مل 321±26 و 719±263، على التوالي، وقد تحققت. بعد 180 يوما، قد انخفض تركيز البلازما متوسط R-بزق في الكلاب الستة إلى 13 نانوجرام/مليلتر. قيمة متوسط تركيز S-بزق كان أعلى من R-بزق في فترة ال 90 يوما الأولى لكنها سقطت بعد ذلك ويمكن لا أن تقدر بدقة عند إسقاط أدناه 20 نانوجرام/مليلتر (الحد الأدنى للمنهجية لهذا صوريا). مراعاة جميع الكلاب، متوسط الحد الأقصى (جكحد أقصى) S-بزق في البلازما خلال الشهور الثلاثة الأولى وكان التركيز أعلى من R-بزق 114.0% (ف < 0.05)، في حين تم الاحتفاظ بها يعني متوسط الوقت (MRT) R-بزق في البلازما أعلى من S-بزق نسبة 96.3 في المائة (ف < 0.05).

الاستنتاجات: المعالجة المشتركة بالبرازكوانتيل نظراً كما أدت إلى صيغة الإفراج عن بطء في الموقع عن طريق الحقن تحت الجلد في تركيزات مبدأ النشط في الكلاب بيغل، التي ينبغي أن تكون قادرة على مقاومة الإصابات الجديدة المشوكة لمدة 6 أشهر على الأقل. الصياغة الجديدة للمعالجة المشتركة بالبرازكوانتيل يمثل طريقة البديلة المحتملة، وتقديم الأدوية ضد عدوى الدودة الشريطية في الكلاب.

Translated from English version into Arabic by selvana, through

TRANSLATORS
WITHOUT BORDERS

犬用吡喹酮缓释制剂：包虫病防控新剂型的研究

姜斌，周晓农，张皓冰，陶奕，霍乐乐，刘妮

**摘要**

**引言：**包虫病是一种全球分布、危害严重的人兽共患寄生虫病。根据 2012 年中国流行病学调查结果，有 2 万名包虫病患者以及 5000 多万人处于感染风险。由于犬是主要的终宿主，中国政府鼓励“月月驱虫，犬犬投药”。然而，在环境恶劣地区很难做到这一点，如青藏高原，很多地方有许多流浪犬。为克服该问题，我们研制了新型吡喹酮缓释制剂，通过对犬皮下注射从而阻断包虫病传播。

**方法：**采用 6 只比格犬进行药代动力学实验，研究了新型吡喹酮缓释制剂中吡喹酮手性对映体的药代动力学立体选择性，如与一种可降解的生物降解聚合物混合的左旋吡喹酮（R-PZQ）和右旋吡喹酮（S-PZQ）。该制剂皮下注射剂量为 100 mg/kg。采用手性高效液相色谱法（HPLC）-高分辨率质谱法（HRMS）测定比格犬血浆中吡喹酮对映体的含量。R-PZQ 和 S-PZQ 的最低检测限分别为 4 ng/ml 和 20 ng/ml。采用药物分析系统（DAS）软件 2.0 的非房室模型对药代动力学参数进行计算。采用 SPSS 19.0 软件进行统计分析，吡喹酮对映体之间采用双侧 t 检验进行统计比较。

**结果：**新型吡喹酮缓释制剂皮下注射 2 小时后，R-PZQ 和 S-PZQ 的最大峰浓度分别为 321±26 和 719±263 ng/ml。180 天后，R-PZQ 的平均血浆浓度下降到 13 ng/ml。在前 90 天，S-PZQ 的平均血药浓度均高于 R-PZQ。但 90 天后，S-PZQ 的平均血药浓度均下降在低于 20 ng/ml （S-PZQ 的最低检测限）。实验中，S-PZQ 的平均最大血药浓度（Cmax）在前 3 个月均显著高于 R-PZQ 的 Cmax 114.0%（$P < 0.05$），而 R-PZQ 的平均驻留时间（MRT）均显著高于 S-PZQ 的 MRT 96.3%（$P < 0.05$）。

**结论：**本项目中新型吡喹酮缓释制剂经皮下注射，在比格犬皮下原位固化缓慢释放活性药物，具备阻断包虫病传染至少 6 个月的潜力。该吡喹酮新剂型为抗犬绦虫感染提供了一种潜在的、可替代的药物治疗方法。

Translated from English version into Chinese by Bin Jiang

**Diffusion lente de praziquantelpour les chiens: présentation d'une nouvelle formule pour le contrôle de l'échinococcose.**

Bin Jiang, Xiao-Nong Zhou, Hao-Bing Zhang, Yi Tao, Le-Le Huo, Ni Liu

**RÉSUMÉ**

**Rappel des faits:** l'échinococcose est une maladie grave, zoonose, parasitaire avec distribution dans le monde entier. Selon une étude épidémiologique de 2012 en Chine, il y a 20 000 patients infectés et plus de 50 millions de personnes à risque. Comme le chien est l'hôte principal et définitif, le gouvernement chinois encourage un traitement mensuel de chaque chien au praziquantel. Cependant, c'est difficile à réaliser dans des zones difficiles sur le plan géographique, tels que le plateau tibétain, où il y a aussi beaucoup de chiens sans propriétaires. Pour résoudre ces problèmes, nous avons étudié l'efficacité de blocage de la transmission par l'intermédiaire d'une formulation à libération lente de praziquantel administré par injection sous-cutanée.

**Méthodes:** L'impact d'une préparation à libération lente de deux pharmacocinétiques stéréosélective praziquantel énantiomères, c'est-à-dire R-(-)-praziquantel (R-PZQ) et S-(+)-le praziquantel (S-PZQ) absorbés dans un polymère biodégradable a été étudié chez des chiens beagle ($N = 6$). La préparation a été administrée par voie sous-cutanée à l'aide d'une dose unique de 100 mg/kg. La sélection par

chiralité,la chromatographie en phase liquide à haute performance (CLHP) et la spectrométrie de masse à haute résolution (SMHR) ont été utilisées pour mesurer les praziquantel énantiomères dans le plasma des chiens. La limite inférieure pour l'estimation des concentrations plasmatiques avec précision pour R-PZQ était de 4 ng/ml et pour S-PZQ 20 ng/ml. Les paramètres pharmacocinétiques ont été calculées par un modèle d'analyse non-compartimentales utilisant le logiciel Drug Analyze System (DAS) 2.0. Le logiciel SPSS 19.0 a été utilisé pour des analyses statistiques, et la comparaison statistique entre les énantiomères a été mesurée grâce à des tests - *t* - bilatéraux.

**Résultats:** deux heures après l'administration, les pics de concentration en R-PZQ et S-PZQ: respectivement 321±26 et 719±263 ng/ml, , ont été atteints. Après 180 jours, la concentration moyenne plasmatique de R-PZQ dans les six chiens a atteint 13 ng/ml. La valeur de la concentration moyenne de S-PZQ a été supérieure à celle des R-PZQ dans la première période de 90 jours mais elle a diminué par la suite et pourrait ne pas être estimée avec précision quand elle chute en dessous de 20 ng/ml (la limite méthodologique la plus basse pour cet énantiomère). En prenant tous les chiens en compte, la concentration moyenne maximale ($C_{max}$) de S-PZQ dans le plasma durant les 3 premiers mois était plus élevée que celle de R-PZQ de 114.0% ($P < 0.05$), alors que la durée de rétention moyenne représentative (MRT) de R-PZQ dans le plasma été plus élevée que celle de S-PZQ de 96.3% ($P < 0.05$).

**Conclusions:** Le praziquantel donné comme une formulation à libération lente *in situ* par voie sous-cutanée a donné lieu à des concentrations du principe actif chez le chien beagle, qui devrait être capable de résister à des nouveaux cas d'infections*Echinococcus* pendant au moins 6 mois. La nouvelle formulation de praziquantel constitue un moyen potentiel et alternatif offrant un traitement contre les infections du ténias chez le chien.

Translated from English version into French by annesophi, through

**Празиквантел замедленного высвобождения для собак: презентация новой формулы для контроля эхинококкоза**

Бинь Цзян, Сяо-Нун Чжоу, Хао-Бин Чжан, И Тао, Лэ-Лэ Хо, Ни Лю

**Аннотация**

**Справочная информация:** Эхинококкоз представляет собой широко распространённое серьёзное зоонозное паразитическое заболевание. По данным эпидемиологического обзора, проведённого в 2012 году, в Китае проживают 20 000 заражённых указанным заболеванием пациентов, и более 50 миллионов человек находятся в группе риска. Поскольку собака является основным, окончательным хозяином, правительство Китая поощряет ежемесячное лечение каждой собаки празиквантелом. Достижение этого, тем не менее, сопряжено с трудностями в географически труднодоступных районах, таких как Тибетское нагорье, которое также является

местом концентрации большого количества бездомных собак. С целью преодоления указанных проблем, мы исследовали на способность блокировки передачи вводимый подкожной инъекцией празиквантел замедленного высвобождения.

**Метод:** Воздействие препарата двух фармакокинетически стереоселективных энантиомеров празиквантела замедленного высвобождения, то есть R-(-)-празиквантела (R-PZQ) и S-(+)-празиквантела (S-PZQ), поглощаемых биоразлагаемыми полимерами, было изучено на собаках породы бигль ($N = 6$). Препарат был введён путём инъекции с использованием разовой дозы-100 мг/кг подкожно. Для измерения энантиомеров празиквантела в плазме крови собак были задействованы хирально-селективная высокоэффективная жидкостная хроматография (ВЭЖХ) и масс-спектрометрия высокого разрешения(HRMS). Нижний оценочный предел концентрации в плазме крови составил для R-PZQ 4 нг/мл и для S-PZQ 20 нг/мл в точности. Расчёт фармакокинетических параметров был произведён путём анализа без использования камерной модели с помощью программного обеспечения Drug Analyze System (DAS) 2.0. Программное обеспечение SPSS 19,0 было применено для статистического анализа, тогда как оценка статистического сравнения энантиомеров была произведена с помощью двустороннего *т*-теста.

**Результаты:** Пик концентрации R-PZQ и S-PZQ: 321±26 и 719±263 ng/ml соответственно был достигнут через два часа после введения препарата. Через 180 дней средняя концентрация R-PZQ в плазме крови шести собак сократилась до 13 нг/мл. За первые 90 дней среднее значение концентрации S-PZQ было выше, чем R-PZQ, потом произошло снижение, которое невозможно было точно оценить после падения показателя ниже 20 нг/мл (нижний методологический предел для данного энантиомера). Принимая во внимание всех собак, средняя концентрация S-PZQ по максимуму ($C_{max}$)в плазме крови за период более 3 месяцев была выше, чем R-PZQ на 114,0% ($P < 0,05$), в то время, как среднее удержание препарата в организме (MRT) по R-PZQ в плазме крови оказалось выше, чем по S-PZQ на 96,3% ($P < 0,05$).

**Выводы:** Введённый местно посредством подкожной инъекции *празиквантел* с формулой замедленного высвобождения привёл к такой концентрации действующего вещества у собак породы бигль, которая должна оказаться способной противостоять новым инфекциям *эхинококкоза* в течение как минимум 6 месяцев. Новая формула празиквантела представляет собой потенциал, альтернативный способ применения лекарственного препарата против заражения ленточным червем у собак.

Translated from English version into Russian by Liudmila Tomanek (nee Volynets), through

TRANSLATORS
WITHOUT BORDERS

**Praziquantel de liberación prolongada para perros: presentación de una nueva fórmula para el control de la equinococosis**

Bin Jiang, Xiao-Nong Zhou, Hao-Bing Zhang, Yi Tao, Le-Le Huo, Ni Liu

**RESUMEN**

**Antecedentes:** La equinococosis es una enfermedad zoonótica parasitaria grave de distribución mundial. Según un estudio epidemiológico del 2012 en China, hay 20 000 pacientes infectados y más de 50 millones de personas en riesgo. Dado que el perro es el principal huésped definitivo, el gobierno de China promueve que todos los perros reciban tratamiento mensual con praziquantel. Sin embargo, esto es difícil de lograr en las áreas geográficas de difícil acceso, como la meseta tibetana, donde también hay muchos perros sin proprietario. Con la finalidad de superar estos problemas, se investigó la capacidad de bloqueo de transmisión de una formula de liberación prolongada de praziquantel administrado por inyección subcutánea.

**Métodos:** Se estudió el impacto de una preparación de liberación prolongada de dos enantiómeros de praziquantel farmacocinéticamente estereoselectivos, es decir, R - (-) - praziquantel (R - PZQ) y S - ( ) - praziquantel (S - PZQ) absorbidos en un polímero biodegradable de perros beagle ($N$ = 6). La preparación se administró por inyección subcutánea utilizando una dosis única de 100 mg / kg. Para medir los enantiómeros de praziquantel en el plasma de los perros, se aplicaron técnicas selectivas de quirales como la cromatografía líquida de alta resolución (HPLC) y la espectrometría de masas de alta resolución (HRMS). El límite inferior para estimar con precisión las concentraciones plasmáticas, fue de 4 ng / ml para R-PZQ y de 20 ng / ml para S-PZQ. Los parámetros farmacocinéticos se calcularon mediante un modelo de análisis no compartimental utilizando el software DAS (Sistema de Análisis de Drogas) 2.0. Para el análisis estadístico se utilizó el software SPSS 19.0, y la comparación estadística entre los enantiómeros se evaluó con la prueba T bilateral .

**Resultados:** Dos horas después de la administración, se alcanzaron las concentraciones máximas de R-PZQ y S-PZQ: 321 ± 26 y 719 ± 263 ng / ml, respectivamente. Después de 180 días, la concentración plasmática promedio de R-PZQ en los seis perros había disminuido a 13 ng / ml. El valor de concentración promedio de S-PZQ fue mayor que el de R-PZQ en el primer período de 90 días, pero disminuyó después y no fue posible estimarlo con precisión cuando bajó por debajo de 20 ng / ml (el límite metodológico más bajo para este enantiómero). Tomando en cuenta todos los perros, la concentración máxima media ($C_{\text{máx}}$) de S-PZQ en plasma durante los primeros 3 meses fue mayor que la de R-PZQ por 114,0% ( $P$ <0,05), mientras que el valor promedio del tiempo de retención (MRT por sus cifras en inglés) de R-PZQ en plasma fue mayor que el de S-PZQ por 96,3% ( $P$ < 0,05).

**Conclusiones:** La aplicación in situ de la fórmula prolongada de praziquantelpor inyección subcutánea dio lugar a concentraciones del principio activo en los perros beagle, que deberían otorgar resistencia a infecciones causadas por *Echinococcus* por al menos 6 meses. La nueva fórmula de praziquantel representa potencialmente una alternativa de presentación del medicamento contra las infecciones de tenia en perros.

Translated from English version into Spanish by Viveka Guzmán, through

TRANSLATORS
WITHOUT BORDERS
